# Effect of Postoperative Adjuvant Radiotherapy on Quality of Life, Anxiety, and Depression in Adult Female Breast Cancer Patients

**DOI:** 10.7759/cureus.36635

**Published:** 2023-03-24

**Authors:** Murtaza Parvizi, Engin Kut, Murat Akyol, Semra Ay

**Affiliations:** 1 Radiation Oncology, Manisa City Hospital, Manisa, TUR; 2 Medical Oncology, Manisa City Hospital, Manisa, TUR; 3 Medical Oncology, İzmir Bakırçay University, İzmir, TUR; 4 Manisa Vocational Health School, Manisa Celal Bayar University, Manisa, TUR

**Keywords:** beck depression inventory (bdi), beck anxiety inventory, eortc qlq-c30, psychiatric morbidity, european organisation for research and treatment of cancer quality of life questionnaire-30, quality-of-life, depression, clinical anxiety, radiotherapy (rt), breast cancer

## Abstract

Aim: This study aims to identify anxiety and depression caused by adjuvant radiotherapy in breast cancer cases to determine the deterioration in the quality of life and investigate the effect of early treatment.

Materials and Methods: In this study, the Beck Depression Inventory, Beck Anxiety Inventory, and European Organisation for Research and Treatment of Cancer Quality of Life Questionnaire-30 (EORTC QLQ-C30) Turkish 3.0 forms were evaluated in 63 breast cancer patients before the start of radiotherapy treatment (T1) and at six weeks after the end of radiotherapy treatment (T2).

Results: A high level of anxiety was detected in 77.8% of patients, and depression was found in 25.4% of patients in T1. When depressive cases were evaluated with EORTC QLQ-C30 scores, the general health status (*p* = 0.043), role function (*p* = 0.027), emotional (*p* < 0.002), cognitive (*p* < 0.001), and social (*p* < 0.0001) scales were statistically lower in T1, whereas pain (*p* = 0.045) and insomnia (*p* < 0.0001) symptoms were higher in T1. Anxiety and EORTC QLQ-C30 scores in terms of emotional function (*p* = 0.015), social function (*p* < 0.003), and symptoms of insomnia (*p* = 0.027) were found to be statistically higher in T1 anxious cases. However, anxiety was detected in only 3% of T2 cases, and no depression was found in any of the cases. Anxiety and EORTC QLQ-C30 scores and symptom scales were evaluated in terms of role function (*p* < 0.0001), emotional (*p* = 0.041) and social scales (*p* = 0.014), fatigue (*p* = 0.028), pain (*p* = 0.033), insomnia (*p* = 0.011), and constipation (*p* < 0.0001); these were found to be statistically significant in T2.

Conclusion: This study revealed that early diagnosis and treatment of anxiety before initiating adjuvant radiotherapy reduces the development of long-term anxiety-related depression in the future. Therefore, it is recommended that patients be evaluated for anxiety and depression before starting adjuvant radiotherapy.

## Introduction

Breast cancer in women can develop at any age after puberty, and recent advances in diagnosis and treatment are promising in terms of the survival of this disease. According to the World Health Organization, 2.3 million women were affected by this disease worldwide in 2020, and 685,000 women died from it [1].

Anxiety is a common reaction to the perception of threats after a cancer diagnosis, such as the loss of bodily functions, change in appearance, deterioration of family structure, and death. In cancer patients, anxiety might last the course of the illness, remarkably influencing the patient’s quality of life (QoL) and frequently coexisting with depression. Anxiety tends to occur or worsen at critical points in the course of disease [2,3]. The coexistence of cancer and depression has been proven with strong evidence, with a 20-50% frequency of depression with solid tumors [4]. Prolonged high levels of mental distress can lead to anxiety, depression, or both in cancer patients. Coexistence of these symptoms is extremely common, with two-thirds of depressed cancer patients also expressing clinically considerable levels of anxiety [5,6]. 

As described in many studies, all cancer patients are not the same. Many variables such as breast cancer, head and neck cancer, etc., age, stage of the disease, diagnosis, and treatment time are among the most important variables affecting the prevalence of depression and should be taken into account in the treatment process of patients. Psychiatric morbidity and other psychiatric events have been evaluated more in breast cancer patients compared to cases with other regions of cancer, where less data are available [7,8]. Inadequate or delayed diagnosis of major depression in cancer patients reduces their QoL, increases the chance of prolonged hospital stay during the treatment process, and leads to high rates of noncompliance over the treatment period [8].

QoL has become an accepted treatment success measure for cancer patients, and an integral part of cancer patient treatment is follow-up management. Advances in early diagnosis and treatment of breast cancer have resulted in patients surviving longer. In addition, because breast cancer affects the identity of women, it is vital to investigate the QoL of women who lose their breasts [9,10]. 

The diagnosis time, early phase of adjuvant therapy, and periods after integration of adjuvant therapy are the transition periods of nonadherence and poor QoL in breast cancer patients. Early termination of treatment due to a decrease in QoL caused by side effects of chemotherapy application has been shown in studies [9,11]. Also, it is known that the end of treatment is extremely stressful for women with breast cancer, especially patients receiving adjuvant chemotherapy and/or radiotherapy treatment [12].

Patients with breast cancer experience physical symptoms and psychosocial problems that negatively affect QoL. To determine QoL, parameters including physical functionality, psychological well-being (e.g., levels of anxiety and depression), and social support are usually considered. This support may vary in the diagnosis and treatment stages of breast cancer cases [9,10].

According to previous studies, the addition of adjuvant radiotherapy to the treatment of breast cancer patients who underwent mastectomy was associated with a higher incidence of psychological morbidities, such as depression and anxiety [9,10,12]. Studies of patients who have undergone breast-conserving therapy (lumpectomy plus breast irradiation) have suggested that this treatment may be associated with an increase in affective symptoms compared to patients who underwent mastectomy or lumpectomy alone [13]. Furthermore, some studies have shown that postoperative radiotherapy treatment has no effect on psychological discomfort, but it has been observed to have significant effects on physical symptoms, particularly fatigue [14].

Therefore, we conducted this study to determine the anxiety and depression caused by adjuvant radiotherapy in breast cancer cases, to determine the deterioration in QoL, and to investigate the effect of early treatment.

## Materials and methods

Study design

This study includes 63 cases of women who underwent an operation (breast-conserving surgery or modified radical mastectomy) and received adjuvant radiotherapy due to breast cancer between May 20, 2016, and October 31, 2017, in Manisa State Hospital’s Radiation Oncology Clinic, Manisa, Türkiye. The Health Sciences Ethics Committee of Manisa Celal Bayar University issued approval for the study (approval number: 727821665/772.02 dated May 18, 2016).

Patients over 18 years of age, pathologically diagnosed with breast cancer, who underwent surgery (breast-conserving surgery or modified radical mastectomy) and received radiotherapy were included in the study. Patients who had more than one primary, were younger than 18 years of age, had no pathological diagnosis of breast cancer, and/or received metastatic or palliative radiotherapy were excluded from the study. The volunteers who participated in the study were female patients who were diagnosed with breast cancer, underwent operations, and indicated postoperative radiotherapy. The data were collected by the primary physician and trained interviewers during face-to-face interviews using a series of predetermined forms in a specified time frame to evaluate patients’ psychological state and QoL. First, patient information and consent forms were filled in, then patient general demographics and clinic information were noted. After, the Beck Depression Inventory (BDI), Beck Anxiety Inventory (BAI), and European Organization for Research and Treatment of Cancer Quality of Life Questionnaire (EORTC QLQ-C30) Turkish 3.0 survey forms were given to the patients to be consecutively filled in.

BDI

The BDI contains 21 variables and is used to determine the presence of depression according to the total value after each variable, which is scored between 0 and 3 (0 = symptom not present; 3 = symptom very intense). The patients completed the BDI with 21 variables (self-report); patients with a Beck depression score of >17 points were accepted as depressed [15].

BAI

The BAI contains 21 items that need to be answered individually, including symptoms such as irritability, dizziness, and inability to relax. The subjects answered this 21-item inquiry form by scoring between 0 and 3 according to their general condition a week ago [16]. The patients completed the BDI with 21 variables (self-report); patients with a Beck depression score of >15 points were accepted as depressed

EORTC QLQ-C30

The EORTC QLQ-C30 is a 30-item cancer-specific questionnaire used in international clinical trials that evaluates the QoL of cancer patients. The EORTC QLQ-C30 form includes five functional scales (physical, role, cognitive, emotional, and social), three symptoms (fatigue, pain, and nausea and vomiting), nine multi-item scales assessing general health and QoL, and several single-symptom scales (e.g., dyspnea, anorexia, sleep disturbance, constipation, and diarrhea). All items are scored on four-point Likert scales, ranging from 1 (no finding) to 4 (very high), except for two items in the global health/QoL scale that use modified seven-point linear analog scales [17].

QoL assessment in cancer patients has gained considerable attention in recent years. For this purpose, EORTC had formed a study group evaluating QoL. One of this group’s major tasks was to develop questionnaires for the assessment of QoL in international clinical trials. More recently, the EORTC QoL study group revised the response categories of the physical functioning scale from a double response to a quadruple response system, the EORTC QLQ-C30, version 3.0.

For all cases included in the study, survey forms were filled in by patients one day before starting radiotherapy (Time 1 = T1) and on the first control day (Time 2 = T2) six weeks after ending it. During the completion of the survey forms, a psychiatrist consultation was requested when necessary, and medical treatment was initiated if deemed necessary by the specialist.

Statistical analyses

The variables were frequency, categorical percentage, mean, and standard deviation. Paired samples t-tests were used to compare categorical variables, the BDI, BAI, and EORTC QLQ-C30 scores between groups. The cut-off value of BDI <17 vs. BDI ≥ 17 and BAI < 15 vs. BAI ≥15 points were used in this study. Statistical analysis of the compared changes of the patients’ scores in the time duration and scales for time change (one day before treatment and six weeks after the treatment follow-up period) was evaluated by one-way analysis of variance (ANOVA). A value of p < 0.05 was considered significant

## Results

The sociodemographic and clinical characteristics of the patients are summarized in Table 1. All cases were female, and their median age was 51.14 ± 9.47 (29-74). Further, 82.5% of patients were married, and the Eastern Cooperative Oncology Group (ECOG) performance status of all patients was 0 or 1. Thirty-eight (60.3%) patients were nonsmokers, and none of them used alcohol. Most patients (71.4%) had breast-conserving surgery. Sixty patients (95.2%) received adjuvant chemotherapy, and three patients (4.8%) received adjuvant postoperative hormonotherapy before starting adjuvant radiotherapy. 

**Table 1:** Some sociodemographic characteristics of cancer patients (n=63) *1 TL = 0.38 USD TL: Turkish lira

Characteristics		
Age (mean ± SD; min–max)	51.14 ± 9.47 (29-74)	
Marital status	n	%
Married	52	82.5
Single (widow, divorced, not married)	11	17.5
Education		
Primary education	5	7.9
High school	54	85.7
University	4	6.3
Monthly income		
TL 0–1500*	26	41.3
TL 1500–2000	21	33.3
TL 2000 and above	16	25.4
Smoker		
Yes	25	39.7
No	38	60.3
Primer surgery		
Breast conservative surgery	45	71.4
Modified radical mastectomy	18	28.6
Disease stage		
Stage I	20	31.7
Stage II	25	39.7
Stage III	18	28.6

According to their BDI and BAI scores, 25.4% of the patients had depression at the T1 time (BDI ≥ 17), whereas none of them reported depression at the T2 period (all patients had BDI < 17). The difference between BDI scores in the T1 and T2 periods was statistically significant (p < 0.0001). In terms of the BAI score, a high level of anxiety was found in 77.8% of the patients at the T1 time (BDI > 15), and this rate was defined as 3.0% during the T2 period (p < 0.0001).

Table 2 shows the patients’ EORTC QLQ-C30 results at two different times, T1 and T2. As seen in Table 2, when the QoL questionnaire evaluation data at the two times of the study were taken into consideration, the subjects’ general health status had better values in the T2 time period, and when they underwent functional and symptomatic evaluation, the T1 evaluation had higher scores. It is observed that the values decreased significantly at T2.

**Table 2:** Comparisons of the EORTC QLQ-C30 scores at two different times Results are denoted as mean ±standart deviation EORTC QLQ-C30: European Organisation for Research and Treatment of Cancer Quality of Life Questionnaire-30; T1: one day before starting radiotherapy; T2: six weeks after ending therapy

EORTC QLQ-C30	Item	T1	T2
Component	No/s	(n= 63)	(n= 63)
Global health status	20 - 30	73 ±12	88 ± 6
Functional scale			
Physical	1 - 5	15 ± 9	10 ± 5
Role	6.7	8 ± 13	0 ± 3
Emotional	21 - 24	13 ± 12	4 ± 6
Cognitive	20 - 25	5 ± 10	2 ± 5
Social	26 - 27	3 ± 8	1 ± 6
Symptom scale/items			
Fatigue	10, 12, 18	19 ± 14	6 ± 10
Nausea/vomiting	14 - 15	5 ± 8	0 ± 0
Pain	9.19	10 ± 14	1 ± 6
Dyspnea	8	3 ± 9	1 ± 5
Insomnia	11	14 ± 23	8 ± 14
Appetite loss	13	6 ± 16	4 ± 11
Constipation	16	1 ± 7	1 ± 7
Diarrhea	17	0 ± 0	0 ± 0
Financial difficulties	28	1 ± 7	0 ± 0

Table 3 summarizes the patients’ BDI, BAI, and EORTC QLQ-C30 scores from both groups at the two time points. The BDI score at T2 (BDI ≥ 17) in Table 3 is empty because at T2 all patients registered as non-depressed. Comparing EORTC QLQ-C30 scores, global health status (p = 0.043), role functioning scale (p = 0.027), emotional scale (p < 0.002), cognitive scale (p < 0.001), and social scale (p < 0.0001) were significantly lower in depressive patients (BDI ≥ 17) at T1. EORTC QLQ-C30 symptom scale scores, including pain (p = 0.045) and insomnia (p < 0.0001), were significantly higher in depressed cases (BDI ≥ 17) at T1. As shown in Table 3, the BAI and EORTC QLQ-C30 scores at T1 and the EORTC QLQ-C30 emotional functioning scale (p = 0.015), social functioning scale (p < 0.003), and symptom scale scores including insomnia (p = 0.027) were found to be significantly higher in anxious cases (BAI ≥ 15) compared to other cases (BAI < 15).

**Table 3:** The comparison of EORTC QLQ-C30 scores and anxiety and depression levels of the patients (n = 63) **Kruskal-Wallis Test EORTC QLQ-C30: European Organisation for Research and Treatment of Cancer Quality of Life Questionnaire-30, BDI: Beck Depression Inventory; BAI: Beck Anxiety Inventory

EORTC QLQ-C30	BDI <17	BDI ≥17				BDI <17		BDI ≥17				BAI <15		BAI ≥15				BAI <15		BAI ≥15	
subscales	(T1)	(T1)				(T2)		(T2)				(T1)		(T1)				(T2)		(T2)	
	mean ± SD	mean ± SD		P٭٭		mean ± SD		mean ± SD		P٭٭		mean ± SD		mean ± SD		P٭٭		mean ± SD		mean ± SD	P٭٭
Global health status	75 ± 11	66 ± 13		0.043		88 ± 6		­none		_		73 ± 12		70 ± 13		0.484		88 ± 6		83 ± 11	0.386
Physical functioning	15 ± 9	16 ± 9		0.993		10 ± 5		­none		_		15 ± 9		15 ± 8		0.904		10 ± 5		13 ± 0	0.424
Role functioning	6 ± 12	14 ± 14		0.027		0 ± 3		none		_		7 ± 13		11 ± 13		0.190		0 ± 2		8 ± 11	0.0001
Emotional functioning	10 ± 10	21 ± 13		0.002		4 ± 6		­none		_		11 ± 10		20 ± 14		0.015		4 ± 5		20 ± 17	0.041
Cognitive functioning	2 ± 7	13 ± 14		0.001		2 ± 5		none		_		5 ± 9		7 ± 12		0.664		2 ± 5		8 ± 11	0.146
Social functioning	0 ± 3	10 ± 14		0.0001		1 ± 6		none		_		1 ± 5		9 ± 14		0.003		1 ± 6		8 ± 11	0.014
Fatigue	18 ± 15	21 ± 13		0.519		6 ± 19		none		_		18 ± 15		25 ± 11		0.077		5 ± 9		22 ± 15	0.028
Nausea and vomiting	4 ± 8	7 ± 10		0.335		1 ± 0		none		_		4 ± 8		8 ± 10		0.166		1 ± 0		1 ± 0	0.100
Pain	8 ± 13	16 ± 16		0.045		1 ± 6		none		_		9 ± 14		14 ± 15		0.237		1 ± 6		8 ± 11	0.033
Dyspnoea	2 ± 8	6 ± 13		0.149		1 ± 5		none		_		2 ± 8		7 ± 14		0.088		1 ± 5		0 ± 0	0.796
Insomnia	7 ± 17	35 ± 28		0.0001		7 ± 14		none		-		11 ± 22		26 ± 26		0.027		7 ± 13		33 ±0	0.011
Appetite	4 ± 11	14 ± 24		0.071		4 ± 11		none		_		6 ± 14		9 ± 20		0.616		3 ± 10		16 ± 23	0.110
Constipation	0 ± 4	4 ± 11		0.095		1 ± 7		none		_		0 ± 4		4 ± 12		0.060		0 ± 4		33 ± 0	0.0001
Diarrhea	0 ± 0	0 ± 0		0.100		0 ± 0		none		_		0 ± 0		0 ± 0		0.100		0 ± 0		0 ± 0	0.100
Financial problems	0 ± 4	4 ± 11		0.095		0 ± 0		none		_		1 ± 6		2 ± 8		0.638		0 ± 0		0 ± 0	0.100

Conversely, in T2, groups were formed according to BAI scores (BAI ≥ 15 and BAI < 15). Cases with lower BAI scores (BAI < 15) had a significantly higher EORTC QLQ-C30 role functioning scale (p < 0.0001), emotional functioning scale (p = 0.041), and social functioning scale (p = 0.014) than the cases with high BAI scores (BAI ≥ 15). In the cases with high BAI scores (BAI ≥ 15), EORTC QLQ-C30 symptom scale scores, such as fatigue (p = 0.028), pain (p = 0.033), insomnia (p = 0.011), and constipation (p < 0.0001), were significantly higher than in the cases with low BAI (BAI < 15) scores.

## Discussion

Anxiety and depression are common complaints in breast cancer patients and affect the continuation of their treatment and their QoL. Our study aimed to investigate the effect of postoperative adjuvant radiotherapy on QoL, anxiety, and depression in women with breast cancer at Manisa state Hospital Radiation Oncology Clinic. And in the study, we found that before radiotherapy patients had high anxiety, depression was more frequent, and their QoL was worse. We found that recognizing signs of anxiety, depression, and poor QoL, and early diagnosis and treatment before initiating adjuvant radiotherapy, may reduce the development of long-term anxiety-related depression in the future. In our study, we evaluated the patients at the beginning of radiotherapy and the sixth week after the end of radiotherapy because radiotherapy has early and late side effects. While late effects, such as cancer secondary to radiotherapy, occur over years, early side effects usually begin with radiotherapy and manifest or disappear by six weeks.

Anxiety can be reflected in the patients as a subjective value of fear in various proportions. Severe levels of anxiety are seen in about 45% of cancer patients at diagnosis [18]. In addition to the development of anxiety, depression symptoms or the coexistence of the two have been frequently reported in cancer patients [5]. In our study, in terms of the BAI score at T1 before starting radiotherapy, 77.8% of the patients had high levels of anxiety (BDI > 15), and 25.4% presented with depression (BDI ≥ 17). According to the findings of this period, when appropriate psychological support or medical treatment was initiated at T2, in terms of the BAI score, only 3% of the patients had a high level of anxiety (BDI > 15), and in the same period (T2) depression (BDI ≥ 17) was not identified. Parallel to the general mental and physical conditions of depressed patients in T1, they have worse general health status, a structure far from role function, emotional function, cognitive function, and social work. It was observed that this situation adversely affected the QoL of the patients in this group. This situation exhibits the importance of psychological support and treatment during oncological treatment. The importance of psychological support and treatment in cancer patients with anxiety or depression has been demonstrated by Kudre [19] and Guarino [20] through a detailed systematic review and meta-analysis. In cancer patients with anxiety and depression, therapy combined with psychological support, drug use, or both may be necessary. Treatment may lead to a decrease in anxiety and consequently an improvement in QoL, but it should be kept in mind that it may be difficult to define anxiety owing to cancer-related symptoms such as lassitude and pain. Similarly, the most frequently identified symptoms in major depression may not reveal complete depression findings in cancer patients.

In solid cancer cases, the relationship between anxiety and depression and the variables of the EORTC QLQ-C30 scale was reported by Tavoli et al. [21]. Additionally, anxiety and depression significantly affect QoL, which was described in many studies [22,23]. Similar to the literature, our study revealed that EORTC QLQ-C30 function scales and global QoL scores were statistically lower in cases with anxiety and depression compared to cases without anxiety or depression. The general health status, role function, and emotional, cognitive, and social function values of the EORTC QLQ-C30 scale were found to be statistically lower in depressive patients during the T1 period. In terms of the EORTC QLQ-C30 symptom scale value, only pain and insomnia were found to be statistically higher in depressive cases during the T1 period. Also, in T1, only EORTC QLQ-C30 function scales and symptoms such as emotional and social function were found to be statistically higher in cases with insomnia and anxiety than in other cases. Again, reconsidering the EORTC QLQ-C30 symptom scales in addition to insomnia in the T2 period, symptoms like fatigue, pain, and constipation were found to significantly affect the QoL of mentioned patients.

The most important finding affecting the QoL during treatment in breast cancer cases is fatigue and nausea due to previous treatments, weakness, and impaired general condition [24,25]. Exercise has been identified as effective at relieving symptoms of lassitude during radiotherapy. Furthermore, it has been observed that patients participating in yoga or aerobic exercise studies such as walking programs along with radiotherapy have better physical functions and less lassitude, anxiety, and insomnia than patients who do not exercise [25]. According to our studies in both periods, insomnia was the most important symptom affecting the QoL in patients with anxiety, and insomnia and pain were among the most important symptoms in patients with depression during the T1 period. Also, symptoms such as fatigue, pain, and insomnia were the main factors affecting the QoL in anxious patients in the T2 period.

Limitations

This study was conducted on breast cancer patients treated in the hospital with radiotherapy. The limitations of the study are that it was carried out in a single center, the follow-up period (six weeks) was short, there were no control groups, and the number of patients was small.

## Conclusions

This study revealed the presence of anxiety/depression and the QoL in women with breast cancer who received adjuvant radiotherapy, before radiotherapy and their relationship with radiotherapy. Early diagnosis and treatment of anxiety before starting adjuvant radiotherapy reduce the development of prolonged anxiety-related depression in the future. Therefore, it is recommended that patients be evaluated for anxiety and depression before starting adjuvant radiotherapy.
